# Gleanings

**Published:** 1898-02

**Authors:** 


### GLEANINGS.

Physical inspection in part by one veterinarian and two laymen inspectors caused the condemnation of 268 head of cattle in Philadelphia, out of some 129,026 head examined—surely an extraordinarily small number, and probably some of these in dairies by tuberculin-test. What about the inspection?

Dr. W. G. Hollingsworth reports successful results with electricity in tongue-lollers, and cites a case in a fast mare that kept her tongue continuously out when in harness. Used a D.D. light current battery, No. 4, home medical pattern, dry-cell of one volt; wires were placed from the carriage-seat to the bit, fastened on each side to the bit. When the tongue was out the current was turned on, and when the tongue was returned to the mouth the current turned off. One week's trial brought the desired result. He reports its successful use in a balky horse.

Dr. J. C. McMeil, of Pittsburg, during the summer of 1897 applied the electrical current in the same manner to a farm-horse that would balk when ploughing. Its use brought out an investigation by a member of the Humane Society, who, after watching its application, highly praised the method as wholly superior to the many methods, often of abuse, in moving balky horses.

The extensive outbreaks of the so-called cerebro-spinal meningitis in Maryland and Illinois during the past fall and winter have caused very great losses to the farmers and others in the districts where the disease prevailed in enzoötic form.

